# A Rapid Method for the Determination of Fucoxanthin in Diatom

**DOI:** 10.3390/md16010033

**Published:** 2018-01-22

**Authors:** Li-Juan Wang, Yong Fan, Ronald L. Parsons, Guang-Rong Hu, Pei-Yu Zhang, Fu-Li Li

**Affiliations:** 1 College of Environmental Science and Engineering, Qingdao University, Qingdao 266071, China; w873644513@163.com; 2 Key Laboratory of Biofuels, Shandong Provincial Key Laboratory of Synthetic Biology, Qingdao Engineering Laboratory of Single Cell Oil, Qingdao Institute of Bioenergy and Bioprocess Technology, Chinese Academy of Sciences, Qingdao 266101, China; hugr@qibebt.ac.cn (G.-R.H.); lifl@qibebt.ac.cn (F.-L.L.); 3 Solix Algredients Inc., 120 Commerce Dr., Ste 4, Fort Collins, CO 80524, USA; ron.parsons@solixalgredients.com

**Keywords:** *Phaeodactylum tricornutum*, fucoxanthin, spectrophotometry, high through-put screening

## Abstract

Fucoxanthin is a natural pigment found in microalgae, especially diatoms and *Chrysophyta*. Recently, it has been shown to have anti-inflammatory, anti-tumor, and anti-obesityactivity in humans. *Phaeodactylum tricornutum* is a diatom with high economic potential due to its high content of fucoxanthin and eicosapentaenoic acid. In order to improve fucoxanthin production, physical and chemical mutagenesis could be applied to generate mutants. An accurate and rapid method to assess the fucoxanthin content is a prerequisite for a high-throughput screen of mutants. In this work, the content of fucoxanthin in *P. tricornutum* was determined using spectrophotometry instead of high performance liquid chromatography (HPLC). This spectrophotometric method is easier and faster than liquid chromatography and the standard error was less than 5% when compared to the HPLC results. Also, this method can be applied to other diatoms, with standard errors of 3–14.6%. It provides a high throughput screening method for microalgae strains producing fucoxanthin.

## 1. Introduction

Fucoxanthin is a carotenoid belonging to the xanthophyll class of carotenoids [1]. In recent years, much work has focused on studying the effect of dietary fucoxanthin and on demonstrating that fucoxanthin can be used as a safe and effective dietary supplement. It has anti-inflammatory, anti-tumor, anti-obesity, anti-diabetes, anti-malarial, and other physiological activities [2,3,4]. Clinical studies have shown that taking fucoxanthin can speed up the metabolism, but will not stimulate the central nervous system [5]. Fucoxanthin is widely found in brown algae and diatoms [6]. In macroalgae, the content of fucoxanthin is about 0.1–1 mg·g^−1^ (dry cell weight). Health products containing fucoxanthin derived from brown algae are already sold on markets. However, the production price is too high to meet the expectations of markets, due to the long growth cycle of macroalgae and the low extraction efficiency [7,8]. In microalgae, especially diatoms, fucoxanthin is one of the main pigments in cells, and accounts for about 1–2.5% of the dry cell weight, which is several fold higher than in macroalgae [9,10]. Fucoxanthin is a major component in the Fucoxanthin-Chlorophyll Protein (FCP) complex [11]. The FCP has the function of trapping light energy and light protection, and plays an important role in photosynthesis in diatoms. In the extraction procedures, the high content of lipid is beneficial to the extraction of fucoxanthin. At present, all over the global market, the pharmaceutical and food from the microalgae are mainly produced by spirulina, *Chlorella*, *Dunaliella*, and *Haematococcus pluvialis*. Due to diverse compounds found in microalgae, there are great potential to develop new products derived from microalgae, such as fucoxanthin, nervonic acid, etc. [12,13,14]. 

*Phaeodactylum tricornutum* is a model species of diatoms. In the early part of 21st century, this species of microalgae has been widely investigated as a potential source for biofuel and/or eicosapentaenoic acid (EPA) with a number of reports discussing the growth of *P. tricornutum* under laboratory or pilot scale [15,16]. *P. tricornutum* grows rapidly in the laboratory and at scale, moreover, the genome and a genetic transformation system have also been published [17,18,19]. Fucoxanthin in the *P. tricornutum* ranges from 15.42–16.51 mg·g^−1^ [20], which is a suitable level for fucoxanthin production. In order to improve the yield of fucoxanthin, physical and chemical mutagenesis could be applied to generate mutants. However, a rapid method to screen for mutants with higher fucoxanthin content is needed to accelerate the discovery of high-content strains. 

At present, the concentration of pigments is mainly determined by high performance liquid chromatography (HPLC) [21,22], which limits the throughput because of extraction time, column time for each run, and requires skilled operators to maintain the equipment. For a high-throughput method, it is essential to simplify the extraction and detection methods, while maintaining accuracy. In plants, plankton and green algae, the Chl *a*, *b*, and total carotenoid contents can be determined by using spectrophotometric methods [23,24,25,26]. However, the reported methods either have complicated extraction methods or are biased towards pigment complexes found in Chlorophytes. In this study, we show that the concentration of fucoxanthin can be assessed using a spectrophotometer measuring values at 663 nm, 445 nm, and 750 nm. This work will not only improve screening efficiency of *P. tricornutum* mutants, but also increase the monitoring efficiency of fucoxanthin content in the cultivation process.

## 2. Results

**Figure 1:**
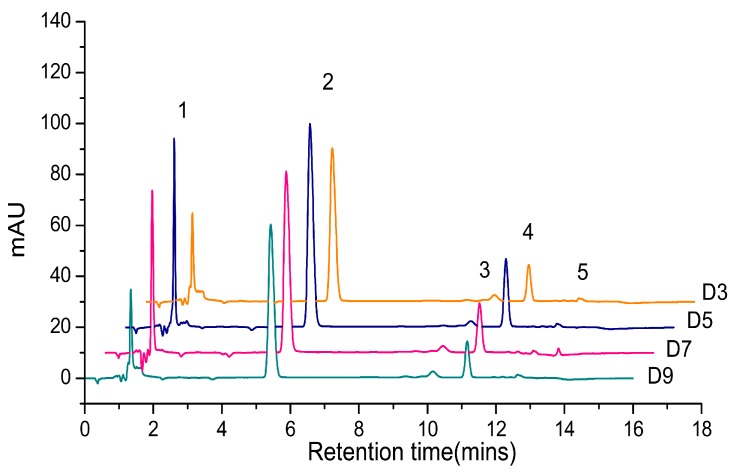
The high performance liquid chromatography (HPLC) chromatogram of total pigments in *P. tricornutum*. Different colored lines represent different culture days. 1: Chl *c*; 2: Fucoxanthin; 3: Diatoxanthin; 4: Chl *a*; 5: β-carotene.

**Figure 2:**
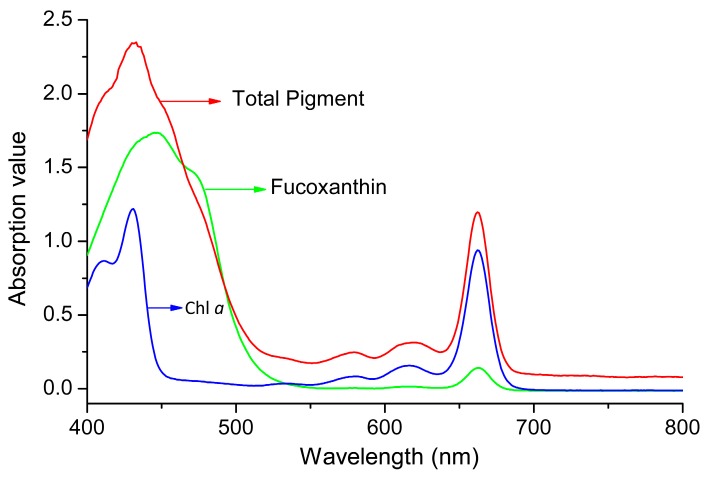
The visible spectrum scan of pigments in *P. tricornutum*. Green line: fucoxanthin; red line: total pigment; blue line: Chl *a*.

**Figure 3:**
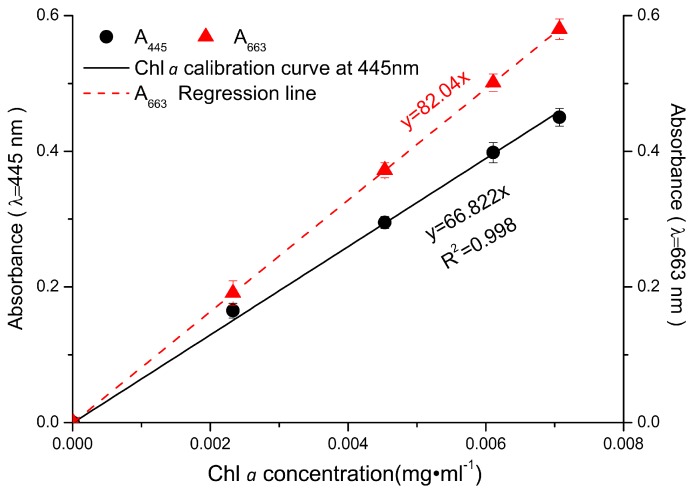
The absorbance values of Chl *a* at 445 nm and 663 nm. Red dots: A_663_ of Chl *a* from tobacco leaf, microalgae *N. oceanica, M. afer*, and *P. tricornutum*, respectively; red line: the line of best fit for the A_663_ values. Black dots and line show the data for the samples at A_445_.

When the total pigments of *P. tricornutum* at different culture stages were quantified by HPLC, there are mainly five product peaks: chlorophyll *c*, fucoxanthin, diatoxanthin, chlorophyll *a*, and β-carotene. Chl *c* levels varied greatly at different culture stages, but Chl *a* and fucoxanthin were relatively stable over the culture (Figure 1). The visible spectrum scan of the total pigment extract contained two main peaks. A broad peak centered at 445 nm and a sharper peak centered at 663 nm. But the purified fucoxanthin exhibits little absorption at 663 nm. However, at 445 nm, the absorption peak has contributions from both fucoxanthin and Chl *a* (Figure 2). Based on the Lambert-Beer law, the content of Chl *a* could be calculated by the absorbance values at 445 nm and 663 nm, respectively. Chl *a* of *P. tricornutum*, *Nannochloropsis oceanica*, *Mychonastes afer*, and tobacco leaf were obtained by thin-layer chromatography (TLC). The Chl *a* concentration was calculated by the absorbance at 663 nm based on Arnon’s method (ε_663 nm_ = 82.04; Formula (2)) [27]. In addition, the light absorption of the samples was measured at 445 nm and a calibration curve was established between the results for the two wavelengths (Figure 3). The extinction coefficient of Chl *a* is 66.8 at 445 nm with the concentration range from 1 to 7 mg·L^−1^.

According to the literature [28,29], the extinction coefficient of fucoxanthin at 449 nm is 1600, with a concentration of 1% (*w*/*v*, g/100 mL). The extinction coefficient at 445 nm was corrected to 156.54 (with a concentration of 1 g·L^−1^) using the purified standard as a sample over the concentration range from 0.005 to 0.05 mg·L^−1^.

According to the additivity law of absorbance, the A_445_ of the pigment extract is the absorbance of fucoxanthin and Chl *a* at 445 nm (Formula (1); *a*_1_ is the extinction coefficient for fucoxanthin; and, *a*_2_ is the extinction coefficient for Chl *a*), because they are the main absorbing components at 445 nm in *P. tricornutum*.

A_445_ = *a*_1_ × *C_fuc_* + *a*_2_ × *C*_Chl *a*_ (1)

The concentration of Chl *a* is the absorption value at 663 nm divided by the extinction coefficient. The Chl *a* absorption value at 445 nm is the extinction coefficient at 445 nm multiplied by the Chl *a* concentration (Formula (2)). Because we desire to calculate the content of fucoxanthin, Formula (2) was rearranged into the form of Formula (3). When the numerical values for the extinction coefficients were placed into Formula (3), we could further simplify the formula to Formula (4). In the process, we transformed C*_fuc_* to C*_fuc_′* so the results of the equation would be in mg·L^−1^.

(2) $$A_{445}=a_{1}\times C_{fuc}+\frac{a_{2}\times A_{663}}{82.04}$$

(3) $$C_{fuc}=\frac{A_{445}}{a_{1}}-\frac{\frac{a_{2}\times A_{663}}{82.04}}{a_{1}}$$

*C_fuc_*′ = 6.39 × A_445_ − 5.18 × A_663_ (4)

Using Formula (4), we calculated the fucoxanthin content of the culture using culture extracted by ethanol and cell debris removed by centrifugation prior to reading the samples in the spectrophotometer. However, this process was not accurate enough (data not shown), so we needed to optimize the formula further.

**Figure 4:**
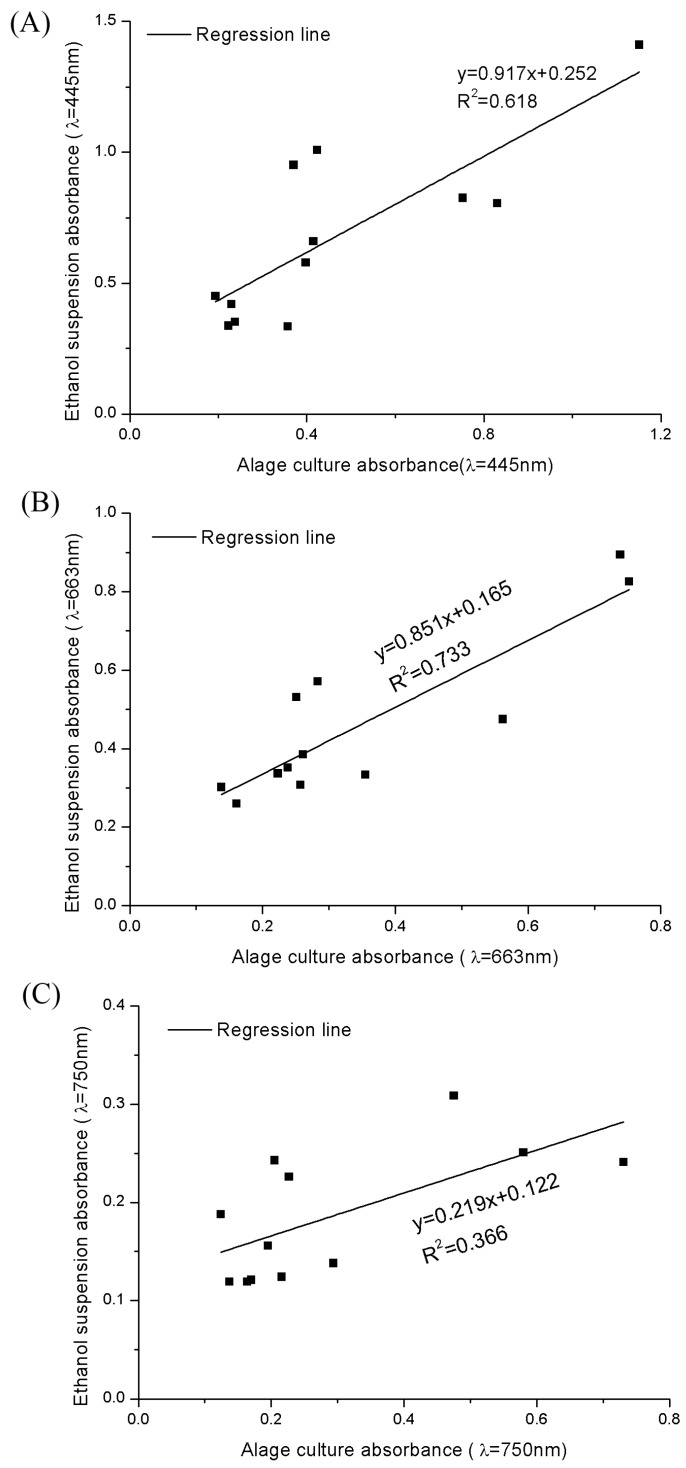
The regression lines of the absorbance values in different wavelength between algae suspension in culture medium (*ASC*) and algae suspension in ethanol (*ASE*). (**A**–**C**) represent the absorbance data at 445 nm, 663 nm and 750 nm, respectively.

We tried to calculate the fucoxanthin concentration in whole cells using the absorbance value of cell culture at 445 nm and 663 nm. However, fucoxanthin is insoluble in the culture medium, and its extinction coefficients in water and ethanol are different. The A_445_ and A_663_ of algal cells suspension in f/2 medium or in ethanol showed no strong correlation coefficients (Figure 4). The absorbance values of algae suspension in culture medium (*ASC*) and algae suspension in ethanol (*ASE*) were collected. The coefficient of determination of the absorbance (R^2^) at 750 nm is 0.366 between *ASC* and *ASE*, they also showed significant difference (*p* = 0.004; Figure 4c). On the other hand, as mentioned before, the A_445_ and A_663_ of the *ASC* also exhibited significant differences from *ASE* (R^2^_445 nm_ = 0.618; *p*_445 nm_ = 0.003; R^2^_663 nm_ = 0.733; *p*_663 nm_ = 0.041; Figure 4a,b). Therefore, if we simplify this method by measuring the data in one situation, we are unable to obtain a correct result.

The previous results suggested that we had to determine the absorbance of algal cells in ethanol at 445 nm and 663 nm with some correction for the interference from other pigments and/or cell debris. Therefore, we needed to remove the “background noise” present in the whole-cell suspensions at 445 nm and 663 nm, which were represented by *n1* and *n2* in the Formula (5), respectively.

*C_fuc_*′ = 6.39 × (A_445_ − *n1*) − 5.18 × (A_663_ − *n2*) (5)

**Figure 5:**
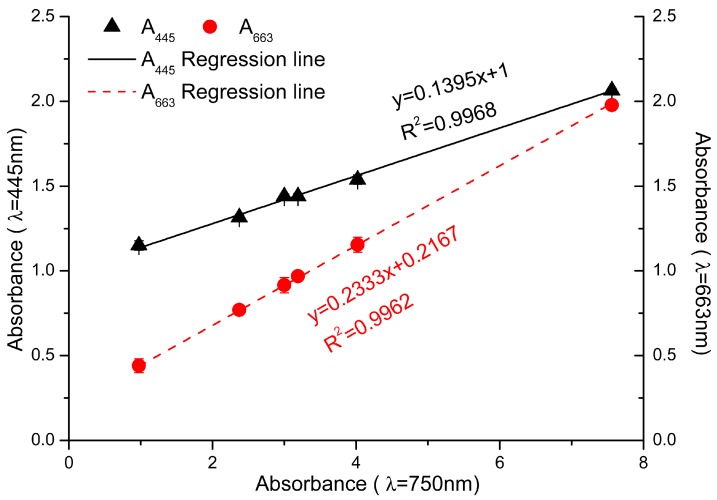
The absorbance value of the residue pigments mixture and cell debris at 445 nm and 663 nm. Black triangles: A_445_ of the mixture; red dots: A_663_ of the mixture. The abscissa showed the absorbance of cell culture at 750 nm. Black and red lines represented the fit curve of the data at different wavelength.

We took an experimental approach to determine the values of *n1* and *n2* in formula 5. In theory, the “background noise” would be a function of the number of cells used to make the measurement. We tested this by determining whether there was a correlation between the number of cells (using A_750_ as a proxy) and cell debris with all of the pigments, except fucoxanthin and Chl *a*. We extracted the pigments from cells, saving the cell debris. After Chl *a* and fucoxanthin were separated out, all of the other pigments (including Chl *c*, diatoxanthin, and β-carotene) and the saved cell debris were resuspended with ethanol and measured at 445 nm and 663 nm. With the absorption value at 750 nm as ordinate, and the absorption value of other pigments and cell debris at 445 nm and 663 nm as abscissa, respectively, the regression curves shown in Figure 5 were obtained.

*n1*(A_445_′) = 0.14 × A_750_ + 1, R^2^ = 0.9968

*n2*(A_663_′) = 0.233 × A_750_ + 0.217, R^2^ = 0.9962

**Figure 6:**
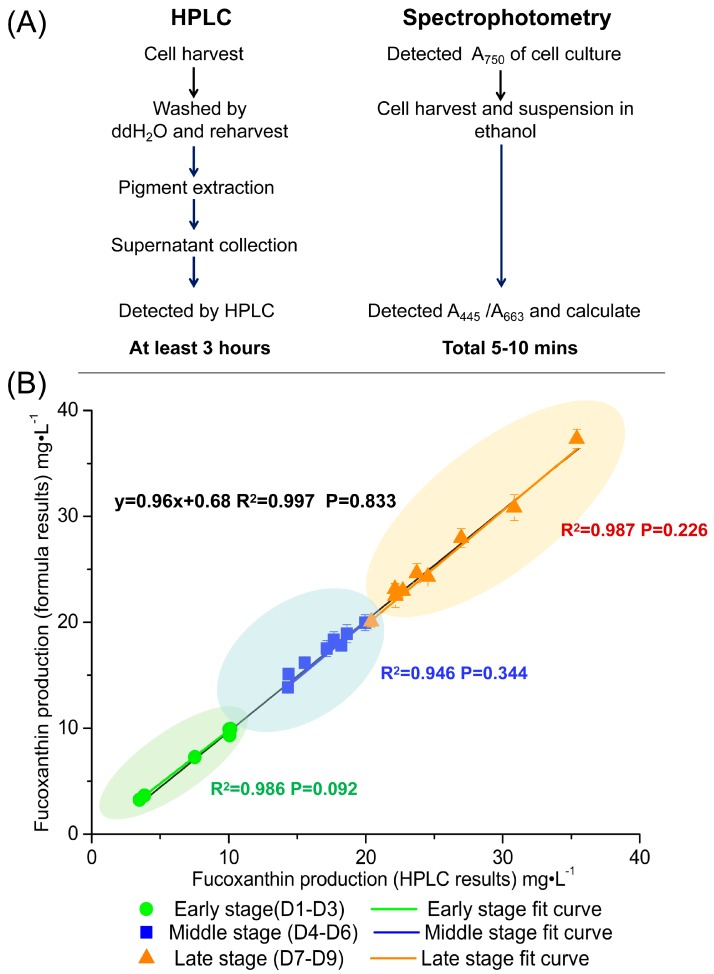
The contrast between HPLC and spectrophotometry. (**A**) The flow-process diagram and time cost of these two methods. (**B**) Spectrophotometric determination is verified at different culture stages. The *x*-axis is fucoxanthin production determined by HPLC, the *y*-axis is fucoxanthin production determined by spectrophotometry. Different colored squares represent different growth stages of the culture. The green area represents the early stage (from 1st day to 3rd day); the blue area represents the middle stage (from 4th day to 6th day); and the orange area represents the late stage (from 7th day to 9th day). The black, green, blue and orange lines represent the curve fit for the total data set, the early stage data set, middle stage data set and late stage data set, respectively. R^2^ is the coefficient of determination. The *p* value is calculated by paired *t*-test. All of the experiments were repeated three times and expressed as mean ± standard deviation (SD).

The equations for the regression lines shown in Figure 5 represent the “background noise” interfering at 445 nm and 663 nm. We can substitute the equations into Formula (5), which results in Formula (6). This can be further simplified to Formula (7). Using Formula (7), the concentration of fucoxanthin in algal cells could be determined through measuring the absorbance of cell culture at 750 nm and algal cell suspension in ethanol at 445 nm and 663 nm (Figure 6A).

*C_fuc_*′ = 6.39 × [A_445_ − (0.14 × A_750_ + 1)] − 5.18 × [A_663_ − (0.233 × A_750_ + 0.217)] (6)

*C_fuc_*′ = 6.39 × A_445_ − 5.18 × A_663_ + 0.312 × A_750_ − 5.27 (7)

In order to verify the correctness of Formula (7), *P. tricornutum* cultures at a cell density of 2 × 10^7^–1 × 10^8^ cells·mL^−1^ at different growth stages were selected for fucoxanthin assay by HPLC and spectrophotometry. Regression analysis and paired *t*-test of these two results were carried out. The statistical analysis of these data derived from above two methods showed in early stage the standard errors were 0.80–4.56%, in middle stage the standard errors were 1.38–4.73%, in late stage the standard errors were 0.23–4.70% (Figure 6B). Therefore, the spectroscopic method has enough accuracy to calculate the fucoxanthin concentration of *P. tricornutum* culture throughout its growth cycle.

**Figure 7:**
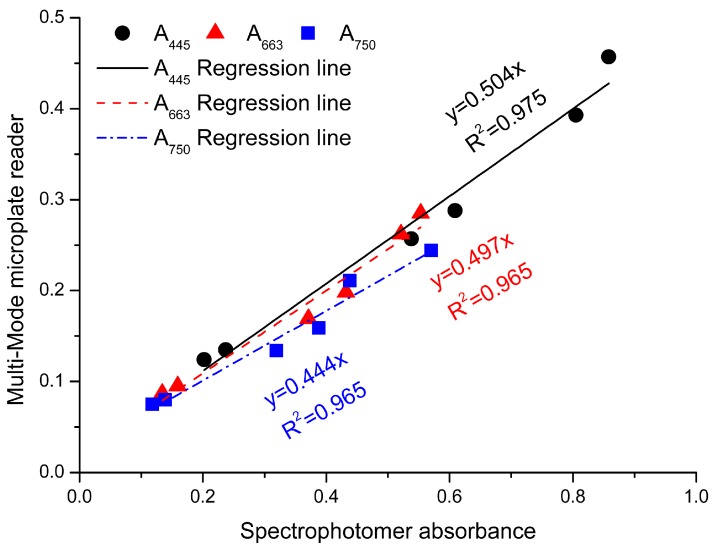
The calibration curves of Multi-Mode microplate reader at different wavelengths. The *x*-axis has the results from the spectrophotometer and the *y*-axis has the results from the Multi-Mode microplate reader. Black dots: absorbance values of *ASE* at 445 nm; red dots: absorbance values of *ASE* at 663 nm; blue dots: absorbance values of *ASC* at 750 nm.

In order to further improve the efficiency of testing fucoxanthin with our spectroscopic method, a multi-mode microplate reader (Synergy™ HT, BioTek, Winooski, VT, USA) was used, in which 96 samples could be analyzed in 10 min for one batch. It was noted that the volume of samples in wells of 96-well plate was 200 μL and the light path was about 0.5 cm. The absorption should be revised by the calibration coefficient of 0.504, 0.497, and 0.444 at A_445_, A_663_, and A_750_, respectively (Figure 7).

## 3. Discussion

Diatoms are a potentially important resource for the production of fucoxanthin. In order to screen for high-fucoxanthin producing strains, a high throughput method for measuring the concentration of fucoxanthin is required. Based on the properties of pigments and statistical analysis, Formula (7) was obtained, which can be efficiently used to calculate the content of fucoxanthin in cultures of *P. tricornutum*.

In developing this method, we wanted to use ethanol to extract the pigment, but the extinction coefficient used for calculating Chl *a* is based on 80% acetone [27,30]. In order to ensure that the extinction coefficient determined in 80% acetone could be used with our method, we compared the absorbance value (at 663 nm) of Chl *a* isolated from different plant or algal material in either 80% acetone or ethanol. The results for the samples in different solvents were analyzed by paired *t*-test, and all of the samples show no significant differences (*p* = 0.063). Based on that result, we were convinced that we could use the classic extinction coefficient in our formulas. 

**Figure 8:**
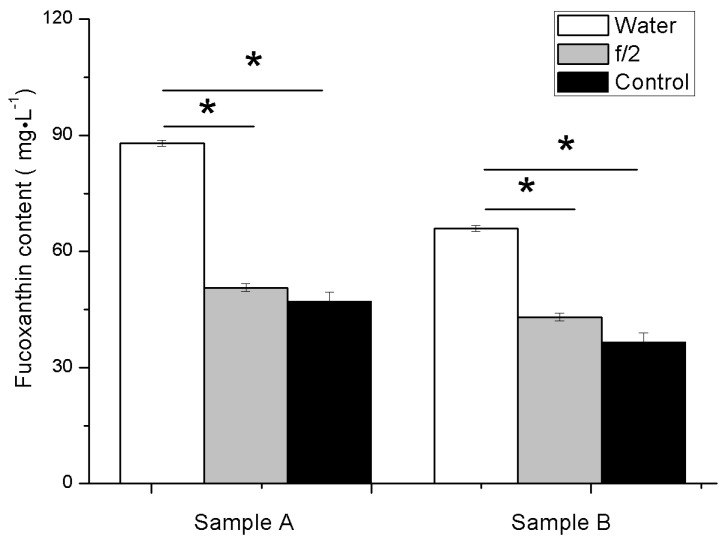
The fucoxanthin concentration of different samples detected by HPLC. Sample A is cells on the 9th day, sample B is cells on the 7th day. Statistically significant differences of fucoxanthin content among different methods are indicated with asterisks above the columns (*p* = 0.05).

When detecting the content of pigment using the HPLC, we found that the extraction efficiency was dependent on whether the cells are washed or not, as well as the wash solution used. We tested three methods to wash the cells after centrifugation prior to extracting the pigments: the first consisted in ddH_2_O, the second in f/2 culture medium, and the third was a control group that was not washed (simply resuspended in the supernatant in the tube after centrifugation). Our results show that washing with ddH_2_O yielded the highest extraction efficiency (Figure 8). This is probably because washing with ddH_2_O started lysing the cells, improving pigment extraction. Based on these results, we used ddH_2_O washed cells to compare the spectrophotometric method against the HPLC method.

Fucoxanthin is widely found in diatoms so we tested our method with two other diatoms: *C. muelleri* and *T. pseudonana* (both centric diatoms). We did a direct test without modifying Formula (7). The results demonstrate that the formula can measure the concentration of fucoxanthin in these two diatom strains with good confidence (Table 1). The standard error for the measurement is larger than what we saw for *P. tricornutum*, presumably due to a lower fucoxanthin content. Therefore, in a rough comparison, this spectroscopic method could be applied to screen other diatom species, as well as being used to quickly detect the fucoxanthin content in production. 

Due to the differences in pigment distribution, cell size, and other cell components at different grow stages (as well as between pennate and centric diatoms), we expected that there would be significant differences within the growth cycle as well as between different species of diatoms that would impact the accuracy of Formula (7). However, we did not see a significant difference among different growth stages of *P. tricornutum* (Figure 6). We did see an increase in the error associated with the centric diatoms. It is possible that developing species-specific measurements of the “background noise” and substituting them in for the *P. tricornutum* values could improve the measurements. In comparison with the HPLC method of fucoxanthin detection, the spectrophotometric method described here simplified the operation process and saved time without markedly affecting accuracy.

## 4. Materials and Methods

### 4.1. Microalgae and Cultivation

Algal strains *P. tricornutum, Mychonastes afer*, and *Nannochloropsis oceanica* IMET1 were provided by the center of BioEnergy Culture Collection (CBECC) in Qingdao Institute of Bioenergy and Bioprocess Technology. *Chaetoceros muelleri* (CCMP 1316) and *Thalassiosira pseudonana* (CCMP 1335) were purchased from National Center for Marine Algae and Microbiota (NCMA, East Boothbay, ME, USA). *P. tricornutum* and *N. oceanica* IMET1 were grown photoautotrophically in modified f/2 medium [31], with increased concentration of sodium nitrate (1 g·L^−1^). *M. afer* was culture in BG-11 medium. Cells were cultured at 23 °C in 100 mL bubble columns (30 cm in height, 4 cm in diameter, 100 mL medium) at a photon flux density of 80 μmol photons·m^−2^·s^−1^ with a 12:12-h light/dark photoperiod.

### 4.2. Extraction and Purification of the Fucoxanthin

The *P. tricornutum* cells in different culture stages were centrifuged at 4000× *g*, then rinsed with ddH_2_O, and recollected by centrifugation. Pellets were suspended in ethanol for pigment extraction (ethanol:algae culture volume = 1:1; *v*/*v*). In agreement with others, we found ethanol to be the most effective solvent in the extraction of fucoxanthin, with the extraction yield being ethanol > acetone > ethyl acetate [20]. The extraction system was incubated at 45 °C for 2 h, and mixed by vortex mixer every half an hour. Finally, the pigment solution was separated by centrifugation at 4000× *g*. The visible spectrum of the pigment solution was obtained by scanning from 400 to 800 nm with a spectrometer (Perkin Elmer UV-VIS Spectrometer Lambda 25, Waltham, MA, USA). 

Purification of the fucoxanthin was carried out by using solid phase extraction (SPE) columns (Agilent Bond Elut HF Mega BE-SI, 5 mg 20 mL, Santa Clara, CA, USA). The pigment extracts were dried under nitrogen and resuspended in the mobile phase (n-hexane:acetone = 6:4) [6]. The total pigments were loaded on the SPE columns, then eluted by the mobile phase. Chl *a* eluted first, followed by fucoxanthin. 

Pigments purity was checked using silica plates (Merck TLC Silica gel 60, Darmstadt, Germany) using hexane:acetone = 6:4 as the mobile phase. The pigment spots were detected visually, scraped from the plate, and then resuspended in ethanol for spectrophotometric analysis.

Quantification of fucoxanthin by HPLC was accomplished using a HITACHI Primaide HPLC system (HITACHI, Tokyo, Japan) with a C_18_ reverse phase column (2.7 μm particle size, 100 × 4.6 mm). The mobile phase consisted of acetonitrile and water with a flow rate of 1 mL·min^−1^. After loading the column with the fucoxanthin extract in ethanol, the mobile phase was an acetonitrile:water solution with the ratio increasing from 80:20 to 100:0 over 8 min, maintained at 100:0 for 3 min, and then decreased back to 80:20 over 5 min. The chromatogram was recorded at 445 nm. Fucoxanthin standards (ChromaDex, fucoxanthin (P), ASB-00006296-010, Irvine, CA, USA) were used for the construction of standard curve in the concentration range of 0.01–1 mg∙mL^−1^.

### 4.3. Spectrophotometric Assay

The extinction coefficient of the Chl *a* at 445 nm was calculated using a published extinction coefficient at 663 nm (ε_663 nm_ = 82.04) [30] using Chl *a* prepared from various sources, including tobacco leaf, *N. oceanica*, and *P. tricornutum*. The absorbance values at 445 nm and 663 nm were measured and a calibration curve was established. This allowed for us to estimate the extinction coefficient of Chl *a* at 445 nm.

For detecting the concentration of fucoxanthin in *P. tricornutum* using a spectrophotometry, the cultures were diluted with culture medium, and the absorbance measured at 750 nm (A_750_ ranges from 0.1 to 0.8). In parallel, a volume of culture was centrifuged and the cells resuspended in an equal volume of ethanol, then the A_445_ and A_663_-values were detected after dilution with ethanol (A_445_ & A_663_ range from 0.2 to 1). Samples were protected from light exposure as much as possible using foil. The cells were suspended in ethanol and analyzed at A_445_ and A_663_ within 5 min. With this data, the concentration of fucoxanthin could be calculated using our formula.

A multi-mode microplate reader (Synergy^TM^ HT, BioTek, Winooski, VT, USA) with 96-well plates was used to study the feasibility of high-throughput analysis following the method described above with the following modifications. The volume of samples in each well was 200 μL. We corrected to a 1 cm path-length using the software provided with the plate reader.

### 4.4. Statistical Analysis

Statistical significance of the values obtained from each experiment was evaluated by regression analysis and paired *t*-test using the software SPSS (version 19.0, IBM, Chicago, IL, USA). All of the experiments were repeated three times. Unless otherwise stated, all data were expressed as mean ± standard deviation (SD). The *p* values of less than 0.05 were considered statistically significant.

## 5. Conclusions

Fucoxanthin is a bioactive substances from marine with high economic value. Using microalgae for the fucoxanthin production enjoy great development and market potential. In this study, we developed an accurate and convenient method to test the concentration of fucoxanthin in diatoms by spectrophotometer. This method not only improve screening efficiency of diatom mutants, but also increase the monitoring efficiency of fucoxanthin content in the cultivation process.

## Figures and Tables

**Table 1:** Comparison of the spectrophotometric methods with HPLC during different culture stages of *Chaetoceros muelleri* and *Thalassiosira pseudonana*.

Culture Stages	*Chaetoceros muelleri* Fucoxanthin Production (mg·L^−1^)			*Thalassiosira pseudonana* Fucoxanthin Production (mg·L^−1^)		
	Spectrophotometry	HPLC	Error (%)	Spectrophotometry	HPLC	Error (%)
Early stage	2.20 ± 0.067	2.5	12.03 ± 2.68	2.27 ± 0.04	2.55	10.98 ± 1.57
Middle stage	2.30 ± 0.043	2.55	5.89 ± 1.69	3.05 ± 0.01	3.38	9.76 ± 0.39
Late stage	9.24 ± 0.042	8.93	3.47 ± 0.47	3.67 ± 0.01	3.51	4.7 ± 0.14

The values are mean ± SD; SD: standard deviation. All experiments were repeated three times.

## References

[1] Gammone M.A., D’Orazio N. (2015). Anti-obesity activity of the marine carotenoid fucoxanthin. Mar. Drugs.

[2] Peng J., Yuan J.P., Wu C.F., Wang J.H. (2011). Fucoxanthin, a marine carotenoid present in brown seaweeds and diatoms: Metabolism and bioactivities relevant to human health. Mar. Drugs.

[3] Woo M.N., Jeon S.M., Kim H.J., Lee M.K., Shin S.K., Shin Y.C., Park Y.B., Choi M.S. (2010). Fucoxanthin supplementation improves plasma and hepatic lipid metabolism and blood glucose concentration in high-fat fed C57BL/6N mice. Chem. Biol. Interact..

[4] Hu X., Li Y., Li C., Fu Y., Cai F., Chen Q., Li D. (2012). Combination of fucoxanthin and conjugated linoleic acid attenuates body weight gain and improves lipid metabolism in high-fat diet-induced obese rats. Arch. Biochem. Biophys..

[5] Maeda H., Hosokawa M., Sashima T., Miyashita K. (2007). Dietary combination of fucoxanthin and fish oil attenuates the weight gain of white adipose tissue and decreases blood glucose in obese/diabetic KK-Ay mice. J. Agric. Food Chem..

[6] Xia S., Wang K., Wan L., Li A., Hu Q., Zhang C. (2013). Production, characterization, and antioxidant activity of fucoxanthin from the marine diatom *Odontella aurita*. Mar. Drugs.

[7] Mori K., Ooi T., Hiraoka M., Oka N., Hamada H., Tamura M., Kusumi T. (2004). Fucoxanthin and its metabolites in edible brown algae cultivated in deep seawater. Mar. Drugs.

[8] Kim S.M., Shang Y.F., Um B.H. (2010). A preparative method for isolation of fucoxanthin from *Eisenia bicyclis* by centrifugal partition chromatography. Phytochem. Anal..

[9] Guo B., Liu B., Yang B., Sun P., Lu X., Liu J., Chen F. (2016). Screening of diatom strains and characterization of *Cyclotella cryptica* as a potential fucoxanthin producer. Mar. Drugs.

[10] Yi Z., Xu M., Magnusdottir M., Zhang Y., Brynjolfsson S., Fu W. (2015). Photo-oxidative stress-driven mutagenesis and adaptive evolution on the marine diatom *Phaeodactylum tricornutum* for enhanced carotenoid accumulation. Mar. Drugs.

[11] Owens T.G. (1986). Light-harvesting function in the diatom *Phaeodactylum tricornutum*: II. Distribution of excitation energy between the photosystems. Plant Physiol..

[12] Yuan C., Zheng Y.L., Zhang W.L., He R., Fan Y., Hu G.R., Li F.L. (2017). Lipid accumulation and anti-rotifer robustness of microalgal strains isolated from eastern china. J. Appl. Phycol..

[13] Yuan C., Liu J.H., Fan Y., Ren X.H., Hu G.R., Li F.L. (2011). *Mychonastes afer*HSO-3-1 as a potential new source of biodiesel. Biotechnol. Biofuels.

[14] Yuan C., Xu K., Sun J., Hu G.R., Li F.L. (2017). Ammonium, Nitrate, and Urea Play Different Roles for Lipid Accumulation in the Nervonic Acid - Producing Microalgae *Mychonastes afer*HSO-3-1. J. Appl. Phycol..

[15] Dambek M., Eilers U., Breitenbach J., Steiger S., Büchel C., Sandmann G. (2012). Biosynthesis of fucoxanthin and diadinoxanthin and function of initial pathway genes in *Phaeodactylum tricornutum*. J. Exp. Bot..

[16] Fernández Sevilla J.M., Cerón García M.C., Sánchez M.A., Belarbi E.H., García C.F., Molina G.E. (2010). Pilot-plant-scale outdoor mixotrophic cultures of *Phaeodactylum tricornutum* using glycerol in vertical bubble column and airlift photobioreactors: Studies in fed-batch mode. Biotechnol. Prog..

[17] Bowler C., Allen A.E., Badger J.H., Grimwood J., Jabbari K., Kuo A., Maheswari U., Martens C., Maumus F., Otillar R.P. (2008). The *Phaeodactylum* genome reveals the evolutionary history of diatom genomes. Nature.

[18] Hamilton M.L., Haslam R.P., Napier J.A., Sayanova O. (2014). Metabolic engineering of *Phaeodactylum tricornutum* for the enhanced accumulation of omega-3 long chain polyunsaturated fatty acids. Metab. Eng..

[19] Zaslavskaia L.A., Lippmeier J.C., Kroth P.G., Grossman A.R., Apt K.E. (2000). Transformation of the diatom *Phaeodactylum tricornutum* (bacillariophyceae) with a variety of selectable marker and reporter genes. J. Phycol..

[20] Kim S.M., Jung Y.J., Kwon O.N., Cha K.H., Um B.H., Chung D., Pan C.H. (2012). A potential commercial source of fucoxanthin extracted from the microalga *Phaeodactylum tricornutum*. Appl. Biochem. Biotechnol..

[21] Schwartz S.J., Woo S.L., Elbe J.H.V. (1981). High-performance liquid chromatography of chlorophylls and their derivatives in fresh and processed spinach. J. Agric. Food Chem..

[22] Braumann T., Grimme L.H. (1981). Reversed-phase high-performance liquid chromatography of chlorophylls and carotenoids. Biochim. Biophys. Acta.

[23] Carreto J.I., Catoggio J.A. (1977). An indirect method for the rapid estimation of carotenoid contents in *Phaeodactylum tricornutum*: Possible application to other marine algae. Mar. Biol..

[24] Carreto J.I., Catoggio J.A. (1976). Variations in pigment contents of the diatom *Phaeodactylum tricornutum* during growth. Mar. Biol..

[25] Parsons T.R., Maita Y., Lalli C.M. (1984). Determination of chlorophylls and total carotenoids: Spectrophotometric method. A Manual of Chemical & Biological Methods for Seawater Analysis.

[26] Dere S., Gunes T., Sivaci R. (1998). Spectrophotometric determination of chlorophyll-A, B and total carotenoid contents of some algae species using different solvents. Turk. J. Bot..

[27] Bruuinsma J. (1963). The quantitative analysis of chlorophylls *a* and *b* in plant extracts. Photochem. Photobiol..

[28] Jeffrey S. (1997). Chlorophyll and carotenoid extinction coefficients. Phytoplankton Pigments in Oceanography: Guidelines to Modern Methods.

[29] Davies B. (1976). Carotenoids. Chemistry and Biochemistry of Plant Pigments.

[30] Arnon D.I. (1949). Copper enzymes in isolated chloroplasts. Polyphenoloxidase in beta vulgaris. Plant Physiol..

[31] Guillard R.R., Ryther J.H. (1962). Studies of marine planktonic diatoms.1. Cyclotella nana hustedt, and detonula confervacea (cleve) gran. Can. J. Microbiol..

